# Special Issue: Radiation Damage in Materials—Helium Effects

**DOI:** 10.3390/ma13092143

**Published:** 2020-05-06

**Authors:** Yongqiang Wang, Khalid Hattar

**Affiliations:** 1 Materials Science and Technology Division, Los Alamos National Laboratory, Los Alamos, NM 87545, USA; 2 Center for Integrated Nanotechnologies, Sandia National Laboratories, Albuquerque, NM 87185, USA

**Keywords:** helium, in situ transmission electron microscopy (TEM), ion beam modification (IBM), extreme environments, molecular dynamic (MD) simulation, nanostructure stability

## Abstract

Despite its scarcity in terrestrial life, helium effects on microstructure evolution and thermo-mechanical properties can have a significant impact on the operation and lifetime of applications, including: advanced structural steels in fast fission reactors, plasma facing and structural materials in fusion devices, spallation neutron target designs, energetic alpha emissions in actinides, helium precipitation in tritium-containing materials, and nuclear waste materials. The small size of a helium atom combined with its near insolubility in almost every solid makes the helium–solid interaction extremely complex over multiple length and time scales. This *Special Issue*, “Radiation Damage in Materials—Helium Effects”, contains review articles and full-length papers on new irradiation material research activities and novel material ideas using experimental and/or modeling approaches. These studies elucidate the interactions of helium with various extreme environments and tailored nanostructures, as well as their impact on microstructural evolution and material properties.

Understanding radiation damage effects in materials, used in various irradiation environments, has been an ongoing challenge since the Manhattan Project, more than 75 years ago. The complexity stems from the fundamental particle–solid interactions and the subsequent damage recovery dynamics after collision cascades, which involves a range of both spatial (Å to m) and temporal (ps to decades) length scales. Adding to this complexity are the transmuted impurities that are unavoidable from accompanying nuclear processes, such as (neutron, alpha) reactions and their interactions with both intrinsic and extrinsic defects through damage recovery and defect evolution processes [1]. Helium (He) is one such impurity that plays an important and unique role in controlling the microstructure and properties of materials. Although abundant in the universe, He is a rare terrestrial resource with even greater scarcity in solid matter due in part to its virtually zero solubility in any material systems [2]. The ultra-low solubility forces He atoms to self-precipitate into small He bubbles that become nucleation sites for further void growth under radiation induced vacancy supersaturations, resulting in material swelling and high temperature He embrittlement, as well as surface blistering under low energy and high flux He bombardment at elevated temperatures. Because it is the large voids (not the small bubbles) that contribute to the detrimental effects, two general approaches have been adopted over the years to mitigate the bubble to void transition [3,4]:

(1) Maximize the critical radius at which bubbles transform into voids, for example, by reducing the vacancy supersaturation; and
(2) Increase the number of stable bubbles by maximizing the number of their nucleation sites (e.g., as in nanoferritic alloys [5,6]), which reduces the He flux to individual bubbles for any given He implantation rate.

Obviously, both approaches do not prevent the formation and eventual linkup of voids, but merely delay it with a hope that other degradation mechanisms become life-limiting. More recently, a totally different approach was proposed, in which carefully engineered semicoherent metal nanolayers were found to alter the fundamental growth trajectory of He-precipitates from the traditional equiaxed growth of 3D nanobubbles into a preferential formation of elongated 1D nanochannels [7]. These 1D He nanochannels that once interconnected to form a 3D network have a potential to outgas He outside of the material in Operando when the material is continuously being implanted with He particles, and thus, effectively reduce the net He particle flux received by the material. During these studies, He implantation has emerged as a useful tool for understanding complex and diverse environments, ranging from solar winds in space [8] to ‘nanofuzz’ formation in fusion energy systems [9].

This *Special Issue*, “Radiation Damage in Materials—Helium Effects”, includes three review articles and nine full length papers (12 publications in total) on new irradiation material research activities and novel material ideas focused on understanding He effects on microstructure evolution and the subsequent properties. The research, originating from 24 different institutions in five different countries (USA, China, UK, Romania, and Japan), utilizes both experimental and modeling approaches to explore the complex interaction of He in a wide range of metallic-based microstructures and compositions (10 in total). These compositions included four ferrous-based systems (Fe, Fe/SiOC, FeCrNi, and Fe12Cr), three copper-based systems (Cu, Cu/V, and Cu/Nb), two tungsten-based systems (W and W-TiC), and palladium (Pd). The impact of the research ranged from fundamental shock wave physics questions such as the role of He impurity on ejecta production in dynamic materials to elucidating the nuclear engineering candidacy of certain alloys and processing routes for advanced fission and fusion reactor concepts. 

The *Special Issue* starts with two modeling papers exploring the fundamental nuances of helium–solid interactions, which in turn permit a greater understanding of the physics and the development of more reliable models predicting the response of He-containing materials. The first article by the team at Los Alamos reviews the rapid developments in the Accelerated Molecular Dynamic (AMD) simulations as applied to the interaction of He in W, which is important for the success of current and future applications in the area of magnetic confinement fusion [10]. This is followed up by a detailed MD simulation by Xu et al., that explores the role of He generation on both grain boundary stability and crack growth in BCC-Fe. This modeling effort also takes advantage of recent advancements in computational code to produce simulated X-Ray Diffraction (XRD) patterns that permit rapid experimental validation [11].

The following six papers nicely demonstrate the complex He defects formed depending greatly on the underlying microstructure and the nuances of the radiation environment. The current understanding of He evolution in solids is well presented in the review “Radiation-Induced Helium Bubbles in Metals” [12]. The nuanced importance of composition and nanostructure for both fusion and Generation IV fission relevant materials are highlighted in the papers by El Atwani et al. exploring equiaxed nanocrystalline W and ultrafine grained W-TiC Alloy [13], by Kim et al.’s study of dual-phase 12Cr oxide-dispersion-strengthened alloy [14], and by Zhang et al. examining swelling and He bubble morphology in a cryogenically treated FeCrNi alloy with martensitic transformation and reversion after He implantation [15]. Two studies in model metal systems (Cu and Pd) noted the impact of radiation environments by comparing various sequential and concurrent heavy ion irradiation and He implantation conditions and by controlling the irradiation temperature. The study on nanocrystalline Cu by the team at Purdue University [16] suggests that He bubbles at grain boundaries and grain interiors may retard grain coarsening. The work on Pd hydriding behavior by the Sandia and University of Huddersfield team [17] utilized hydrogen over-pressure during in situ TEM observation in order to effectively mimic tritium-decay-induced He-3 precipitates in Pd. In both model and application alloys, it is clear that the evolution of He can vary greatly as a function of alloy composition and microstructure, as well as the details of the radiation environment. 

Utilizing the understanding of He evolution at the time, it was proposed and demonstrated in Cu/Nb in 2005 that nanolayered materials with tailored layer thickness and interface structures could greatly decrease the large scale and catastrophic failure in He implanted metals [18]. In the last 15 years, the type of interfaces and layer thickness that have been He implanted has exponentially expanded with a range of engineered microstructures. These research activities are fortunately reviewed in “Interface Effects on He Ion Irradiation in Nanostructured Materials” [19]. This field continues to grow with new results from Chen et al. showing a clever triple layer approach to demonstrate the difference between Cu/V and Cu/Nb interfaces vacancy sink efficiency [20]. Pushing the community away from just metallic nanolayers, the team from the University of Nebraska and Texas A&M University has explored the He evolution in a ceramic/metal (SiOC/Crystalline Fe) nanolayer system [21]. These combined studies show the rapid growth in the study of nanolayers for radiation tolerance and the exciting new directions that are still left to be explored.

The final paper in this *Special Issue* by S. Fensin et al. deviated somewhat from the traditional He effects in materials, where He-induced defects affect microstructural evolution, which further impacts material properties and performance. Instead, this paper used a clever experimental design, utilizing the Richtmyer–Meshkov Instability (RMI) technique to determine “The Role of Helium on Ejecta Production in Copper” [22] and demonstrated a new area of interest in dynamic materials research, where He-doped microstructures are found to directly influence the surface material ejection behavior under shock wave extreme conditions.

Taken together, these studies show a vibrant and still evolving simulation and experimental research community exploring the unique impact He can have on solid matrixes. Despite the scarcity of He on earth, we expect studies exploring the interaction of He in matter will increase as the accessibility of He implantation capabilities via the He Ion Microscope (HIM) [23] becomes a common commercial tool at most research institutes, while simultaneously, the demand for such studies from advanced fission and fusion nuclear reactor, space exploration, actinide research, and nuclear waste communities continues to increase.
